# Factors Associated with Risky Sexual Practices among Female Sex Workers in Karnataka, India

**DOI:** 10.1371/journal.pone.0062167

**Published:** 2013-04-18

**Authors:** Bidhubhusan Mahapatra, Catherine M. Lowndes, Sanjay Kumar Mohanty, Kaveri Gurav, Banadakoppa M. Ramesh, Stephen Moses, Reynold Washington, Michel Alary

**Affiliations:** 1 Population Council, New Delhi, India; 2 Health Protection Services–Colindale, Health Protection Agency, London, United Kingdom; 3 International Institute for Population Sciences, Mumbai, India; 4 Karnataka Health Promotion Trust, Bengaluru, India; 5 Department of Community Health Sciences, University of Manitoba, Winnipeg, Canada; 6 Departments of Medical Microbiology and Medicine, University of Manitoba, Winnipeg, Canada; 7 St. John's Medical College and Hospital, Bengaluru, India; 8 Department of Preventive and Social Medicine, Laval University, Québec City, Canada; UCL Institute of Child Health, University College London, United Kingdom of America

## Abstract

**Introduction:**

The objectives of this study are to develop a summary measure of risky sexual practice and examine the factors associated with this among female sex workers (FSWs) in Karnataka, India.

**Materials and Methods:**

Data were drawn from special behavioral surveys (SBS) conducted in 2007 among 577 FSWs in two districts of Karnataka, India: Belgaum and Bangalore. FSWs were recruited using the two-stage probability sampling design. FSWs' sexual practice was considered risky if they reported inconsistent condom use with any sexual partner and reported experience of one of the following vulnerabilities to HIV risk: anal sex, alcohol consumption prior to sex and concurrent sexual relationships.

**Results:**

About 51% of FSWs had engaged in risky sexual practice. The odds of engaging in risky sex were higher among FSWs who were older (35+ years) than younger (18–25 years) (58% vs. 45%, Adjusted Odds Ratio (AOR): 2.0, 95% confidence interval (CI): 1.2–3.4), who were currently married than never married (61% vs. 51%, AOR: 4.8, 95% CI: 2.5–9.3), who were in sex work for 10+ years than those who were in sex work for less than five years (66% vs. 39%, AOR: 2.6, 95% CI: 1.6–4.2), and who had sex with 3+ clients/day than those who had sex with fewer clients (67% vs. 38%, AOR: 3.7, 95% CI:2.5–5.5).

**Conclusion:**

FSWs who are older, currently married, practicing sex work for longer duration and with higher clientele were more likely to engage in risky sexual practices. HIV prevention programs should develop strategies to reach these most-at risk group of FSWs to optimize the effectiveness of such programs.

## Introduction

HIV prevention programs in India as well as in other countries have implemented various strategies among female sex workers (FSWs) to improve their safe sex behavior. Promoting correct and consistent condom use in all sex acts has been the core of these strategies. However, a growing body of literature suggests that in addition to condom use, practices such as anal sex and alcohol consumption, and the nature of sexual partnerships (single, sequential, multiple or concurrent multiple relationships), are factors that also need attention [1]–[6]. Empirical research suggests that, the practice of anal sex, even with a condom, can increase the chances of getting sexually transmitted infections (STIs) and/or HIV [7]–[10]. Similarly, reports from scientific research indicate a positive association between alcohol consumption and HIV risk [7], [11]–[15]. Moreover, FSWs who consume alcohol are more likely to engage in anal sex than those who do not [10], [16]. Recent advances in HIV research have also highlighted concurrent sexual relationship as another important factor in increasing the rate of infection in a population [5], [6], [17]. Therefore, HIV prevention programs need to develop strategies to address these vulnerabilities among FSWs, with a focus on safe sex behavior.

Empirically, studies have extensively examined FSWs’ socio-demographic characteristics that are associated with inconsistent condom use, anal sex, alcohol consumption prior to sex, and concurrent relationships [10], [14]–[16], [18]–[23]. Based on these findings, program implementers have devised behavior change communication strategies to reach different groups of sex workers. Though this approach of identifying at-risk groups of FSWs has been useful in designing program strategies, there is a need to move a step ahead and identify groups that are most vulnerable to HIV risk. Furthermore, identifying groups of FSWs separately for each of these domains sexual practices may not be advantageous, as at-risk groups identified for inconsistent condom use may not practice anal sex. Moreover, a study among FSWs in India suggests that the practice of anal sex is not associated with inconsistent condom use with clients [10] and hence, developing strategies for each component may not be ideal and can be complex in the long run. In such a scenario, to identify groups that are more vulnerable than others, one needs to adopt a more holistic approach by considering different dimensions of HIV risk behavior and vulnerability. Therefore, this study proposes a summary measure of risky sexual practices among FSWs by considering both measures of behavior and vulnerabilities related to HIV risk. The specific objectives of this study are to develop a summary measure of FSWs’ risky sexual practice and examine the socio-demographic and sex work related characteristics of FSWs that are associated with risky sexual practice in the state of Karnataka, India.

## Materials and Methods

### Study setting

This study is focused in the districts of Belgaum and Bangalore (also known as Bangalore Urban) of Karnataka, which is located in the southern part of India and recognized as a high priority state for HIV prevention by the National AIDS Control Program of India [24]. These districts were selected based on the socio-cultural setting and size of the FSW population. The district of Bangalore is entirely urban with an estimated 19000 FSWs, whereas Belgaum district is semi-urban with only 11000 FSWs [25], [26]. Bangalore district has a population of 9.6 million, of whom 89% are literate [27]. In Bangalore, HIV prevalence among women attending antenatal care clinics (ANC) is 0.8% and among FSWs it is 10% [26]. According to the 2011 Census of India, Belgaum had a population of 4.8 million, with a literacy rate of 74% [27]. In Belgaum HIV prevalence among women attending ANC is 1.5% and among FSWs it is 16% [25].

### Data

Data were drawn from special behavioral surveys (SBS) conducted in 2007 among 577 sex workers in the two study districts: Belgaum and Bangalore. FSWs who were 18 years or older and had sex in exchange for cash or kind in the one month prior to the survey were eligible to participate in the survey. The survey was implemented by the Centre Hospitalier Affilié universitaire de Quebec (CHA) research, monitoring and evaluation, India in collaboration with the Institute of Population Health and Clinical Research (IPHCR), St John’s Medical College, the Karnataka Health Promotion Trust (KHPT), Bangalore, India, and the University of Manitoba, Winnipeg, Canada.

A probability sampling method was employed; samples were drawn using a two-stage sampling design. In the first stage hotspots where FSWs congregate to solicit clients such as streets, brothels, parks, cinema halls and homes were selected. In the second stage respondents were selected from selected hotspots. The sampling frame for this survey was developed by the survey research team with the help of local non-governmental organizations (NGOs) who were implementing the HIV prevention program among FSWs in the study districts. For each hotspot, data were gathered on the number of FSWs present, segregated by the time slot when sex work is undertaken (e.g. 0900–1500 hours, 1500–1900 hours, etc.) and by typology of sex work (home-based, brothel-based and street-based).

The typology of the hotspot was considered as a stratification variable. The number of interviews to be conducted in each typology was allocated proportionately according to its size. A fixed number of hotspots from each typology were selected. Different sampling approaches were adopted to select hotspots in non-street (home and brothel) and street-based settings as the nature of sex work in these settings are different. In non-street-based sex work settings, FSWs are available at any time of the day and hence, the risk profile of FSWs would not vary over the day. However, availability of FSWs in street-based hotspot depends on time and day and hence, the risk profile of FSWs available at a given time point is a function of time and day [28]. Keeping this in mind, home- and brothel-based hotspots were selected using the conventional cluster sampling approach where the first hotspot was selected using a random number and subsequent hotspots were selected using a sampling interval (total number of clusters divided by the number of clusters to be selected). For selection of street-based hotspots, time–location cluster sampling was used, where a hotspot was replicated multiple times to form a cluster for each time slot when FSWs congregate at the hotspot. The survey team had a flexibility of visiting a non-street-based hotspot at any time of the day; whereas in time location clusters, the visit was restricted to the time and day linked to the selected cluster. In the second stage, FSWs were randomly selected from the selected hotspots.

A target sample size of 200 FSWs in Belgaum and 400 in Bangalore was determined. At the end of the survey, a sample of 208 FSWs in Belgaum and 369 in Bangalore was achieved. Sample weights were calculated to account for the unequal selection probability of respondents and non-response rates within each hotspot. The survey instrument was developed in English and translated into Kannada, the local language of Karnataka. The translated forms were reviewed by study investigators fluent in both English and Kannada. The questions asked in the survey instrument were taken from previous research studies conducted in India among sex workers [14], [29], [30]. Trained investigators with verbal and written skills in Kannada conducted face-to-face interviews.

### Ethics statement

The procedures and consent process for this study were reviewed and approved by the institutional review boards of CHA, the University of Manitoba and St. John’s Medical College. In accordance with the protocol, a comprehensive informed consent process was followed and no names or identifying information were recorded. Before the start of the interview the investigator explained to all the eligible respondents the harms and benefits associated with their participation in the survey. Eligible respondents who provided verbal consent to participate were interviewed. Only verbal consent was taken due to the low level of literacy in the study population. The research investigator explained the consent form in the presence of a peer educator who was working with the organization implementing the HIV prevention program in the area. The investigator signed the consent form after taking the consent of the participant. These forms were stored in a location which was accessible only to the principal investigators of the study. Further, interviews were conducted in locations where women were comfortable and their privacy was assured.

### Measures

The key outcome measure in this study is FSWs’ engagement in risky sexual practice. A summary measure of risky sexual practice was constructed by considering four different aspects of FSWs’ HIV risk behaviors and vulnerability. FSWs’ sexual behavior was considered risky if they reported inconsistent condom use with any sexual partner and reported experience of one of the following HIV-related vulnerability: anal sex, alcohol consumption prior to sex and concurrent sexual relationships. This measure takes into account both FSWs’ risk behavior and vulnerability towards HIV risk.

Inconsistent condom use was assessed based on single item questions asked separately for each type of sexual partner (occasional clients, repeat clients, regular partners and non-paying non-regular partners). FSWs were asked about the frequency of condom use for each type of partner with response options of ‘always’, ‘most of the time’, ‘sometimes’ or ‘never’. FSWs who reported having always used a condom with a specific type of sexual partner were considered as using condoms consistently with that type of partner; otherwise considered as using condoms inconsistently. FSWs who reported inconsistent condom use with any partner were categorized as inconsistent condom users; else categorized as consistent condom users. Similarly, single item questions on anal sex and alcohol consumption prior to sex were asked with dichotomized response categories.

Concurrency was measured based on the length of FSWs’ relationships with recent repeat clients and non-paying non-regular partners. Information on length of relationship was collected for three recent repeat clients and non-paying non-regular partners. For each partner, the timing of first sex and last sex was collected. The first step in identifying concurrency involved constructing a timeline of partnerships for each respondent based on the reported start and end dates of sexual contact (the unit of duration was in weeks). Next, concurrency was calculated based on the partnership timelines. A sex worker was considered to have concurrent sexual relationships if the period of relationship with one sexual partner overlapped with at least another sexual partner. As the duration of relationships was measured in weeks, if one partnership ended and another began in the same week, concurrency could not be determined. In such cases, conservatively the respondent was assumed to have a concurrent relationship.

Information on socio-demographic and sex work related characteristics like age (categorized into three categories: 18–24/25–34/35+ years), educational status (no formal education/some formal education), marital status (currently married/formerly married/never married or Devadasi, a traditional form of sex work prevailing in northern Karnataka [25]), income other than sex work (no/yes), residential status (local/non-local, local refers to being a native of the study district), duration of sex work (categorized into three categories: <5/5–9/10+ years), number of clients per day (grouped into two categories: <3/3+), place of solicitation (home-based/brothel-based/street-based) and nature of solicitation (independently/with the help of brokers/pimps) were collected using single item questions. These variables were used as covariates in the multiple logistic regression analyses while predicting risky sexual practice among FSWs.

### Statistical analyses

Univariate, bivariate and multivariate analyses were performed. Univariate analysis was used to describe the profile of the study population. Bivariate analysis was used to present the prevalence of outcome measures (inconsistent condom use, anal sex, alcohol consumption prior to sex, concurrent relationships and risky sexual practice) by FSWs’ socio-demographic and sex work related characteristics. The strength of association of socio-demographic and sex work related characteristics with outcome measures was measured using Pearson’s Chi-square test. A multiple logistic regression model was fitted to examine the correlates of risky sexual practice. Results were presented in the form of percentages (unadjusted), adjusted odds ratios (AOR) and their corresponding 95% confidence interval (CI). All the analyses were carried out using STATA version 12.1 (StataCorp., College Station, TX, USA).

## Results

Sex workers who participated in the survey were, on average, 31 years old (standard deviation (SD): 7 years) and had been practicing sex work for six years (SD: 5.9 years) (Table 1). More than two-thirds had no formal education (71%), earned income only from sex work (70%), were non-native to the study district (70%), and about one-quarter (26%) were currently married. Public places were the primary place of solicitation for the majority of respondents; about 62% of FSWs were street-based as compared to only 17% and 21% -who solicited in home- and brothel-based settings respectively.

**Table 1:** Socio-demographic and sex work related characteristics of female sex workers, Karnataka, India, 2007.

Background Characteristics	% or Mean (SD)*	Number$
**Age (in years)**		
< 25	20.5	120
25–34	40.4	250
35+	39.1	207
Mean (SD)*	30.8 (7.0)	
**Educational status**		
No formal education	71.2	389
Formal education	28.8	188
**Marital status**		
Currently married	25.9	173
Formerly married	56.0	290
Never married/Devadasi^1^	18.2	114
**Income beside sex work**		
No	69.9	380
Yes	30.1	197
**Residential status**		
Local	32.2	217
Non-local	67.8	359
**Duration of sex work (in years)**		
<5	46.3	288
5–9	27.3	156
10+	26.4	133
Mean (SD)*	6.4 (5.9)	
**Number of clients per day**		
<3	55.3	333
3+	44.7	244
**Place of solicitation**		
Home-based	16.8	152
Brothel-based	21.3	146
Street-based	61.9	279
**Nature of solicitation**		
Independently	72.9	385
Through brokers/pimps	27.1	190
**Total**	100.0	577

* SD: Standard Deviation.

$ Unweighted numbers.

1 A traditional form of sex work where females are married to God in childhood and at puberty they start selling sex.

Table 2 provides data on the prevalence of inconsistent condom use, concurrent sexual relationships, anal sex and alcohol consumption prior to sex. About 71% of FSWs reported inconsistent condom use with any client, 32% reported concurrent sexual relationships, 12% had engaged in anal sex and 54% reported consuming alcohol before sex. Compared to FSWs from Bangalore, those from Belgaum district reported higher inconsistent condom use (68% vs. 75%, P = 0.069), anal sex practice (8% vs. 18%, P<0.001) and alcohol consumption prior to sex (47% vs. 65%, P<0.001). Higher inconsistent condom use was reported by FSWs who were in the middle age group (25–34 years) (78%), currently married (94%), and who had sex with three or more clients a day (80%) as compared to their counterparts. Concurrent sexual relationships were higher among FSWs who were young (18–25 years) (42%), those in sex work for 10 years or more (42%) and had sex with three or more clients a day (46%) than their respective counterparts. The practice of anal sex was reported higher by FSWs who were local to the place of interview than those who were not (16% vs. 10%), who were in sex work for 10 years or more than those who were working for less than five years (42% vs. 29%), and those who had three or more clients a day than those who had fewer clients (46% vs. 21%). Consumption of alcohol before sex was positively associated with age, duration of sex work, and number of clients.

**Table 2:** Percent of female sex workers reporting unprotected sex, concurrent sexual relationships, anal sex and consumption of alcohol prior to sex in Karnataka, India, 2007.

Background Characteristics	Number of FSWs$	Inconsistent condom use with any partner	Concurrent sexual relationships	Anal sex	Alcohol prior to sex
**Age (in years)**		P = 0.008	P = 0.041	P = 0.213	P<0.001
< 25	120	62.1	42.1	10.6	33.1
25–34	250	77.5	29.8	14.8	52.6
35+	207	69.4	29.9	9.9	66.5
**Educational status**		P = 0.578	P = 0.560	P = 0.179	P<0.001
No formal education	389	71.8	31.6	10.9	60.5
Formal education	188	69.6	34.2	14.9	38.0
**Marital status**		P<0.001	P = 0.699	P = 0.898	P = 0.110
Currently married	173	93.6	30.0	11.7	47.2
Formerly married	290	63.5	32.4	11.9	55.7
Never married/Devadasi	114	62.8	35.4	12.9	58.8
**Income beside sex work**		P = 0.898	P = 0.069	P = 0.275	P = 0.010
No	380	71.2	34.7	11.1	57.6
Yes	197	70.9	26.9	14.3	45.9
**Residential status**		P = 0.635	P = 0.428	P = 0.041	P = 0.821
Local	217	72.3	34.8	16.1	54.7
Non-local	359	70.5	31.3	10.2	53.5
**Duration of sex work (in years)**		P = 0.207	P = 0.012	P = 0.005	P<0.001
<5	288	68.2	29.1	7.5	37.8
5–9	156	71.4	28.4	14.1	60.9
10+	133	76.1	42.3	17.9	75.4
**Number of clients per day**		P<0.001	P<0.001	P<0.001	P<0.001
<3	333	64.4	21.0	7.2	42.8
3+	244	79.5	46.4	18.0	67.9
**Place of solicitation**		P = 0.070	P = 0.349	P = 0.727	P<0.001
Home-based	152	78.6	36.8	10.1	52.2
Brothel-based	146	64.2	35.1	13.9	71.3
Street-based	279	71.5	30.2	11.9	48.6
**Nature of solicitation**		P = 0.190	P = 0.728	P = 0.173	P = 0.003
Independently	385	72.8	32.9	13.2	50.0
Through brokers/pimps	190	67.1	31.1	9.1	64.4
**Study district**		P = 0.069	P = 0.407	P<0.001	P<0.001
Belgaum	208	75.4	34.3	17.9	65.1
Bangalore	369	68.3	31.0	8.1	46.5
**Total**	577	71.2	32.4	12.0	54.0

**Note:** The P-values were arrived at using Pearson’s Chi-square test and indicates the strength of association between background characteristics and HIV-related sexual risk factors.

$ Unweighted numbers.

As seen in Table 3, about half of the sex workers (51%) had engaged in risky sexual practices; those interviewed in Belgaum were two times more likely to engage in risky sexual practices than those interviewed in Bangalore (64% vs. 42%, AOR: 2.3, 95% CI: 1.4–3.9). The odds of engaging in risky sexual practices were higher among FSWs who were 35 years or older than who were less than 25 years (58% vs. 45%, AOR: 2.0, 95% CI: 1.2–3.4), those who were currently married than those who were never married (61% vs. 51%, AOR: 4.8, 95% CI: 2.5–9.3), those who were in sex work for 10 years or more as compared to those who were in sex work for less than five years (66% vs. 39%, AOR: 2.6, 95% CI: 1.6–4.2), and those who had sex with three or more clients a day than who had sex with fewer clients (67% vs. 38%, AOR: 3.7, 95% CI:2.5–5.5).

**Table 3:** Unadjusted percent and adjusted odds ratio and corresponding 95% confidence interval predicting the odds of risky sex among female sex workers with their socio-demographic and behavioral characteristics as predictor variables in Karnataka, India, 2007

Background characteristics	Number of FSWs$	% FSWs who reported risky sex^1^	AOR (95% CI)^2^
**Age**			
< 25	120	45.4	Referent
25–34	250	46.2	0.9 (0.6–1.6)
35+	207	57.6	2.0 (1.2–3.4)
**Educational status**			
No formal education	389	52.6	1.1 (0.7–1.8)
Formal education	188	45.3	Referent
**Marital status**			
Currently married	173	60.6	4.8 (2.5–9.3)
Formerly married	290	45.7	1.6 (0.9–2.8)
Never married/Devadasi	114	51.0	Referent
**Income beside sex work**			
No	380	51.9	1.4 (0.9–2.1)
Yes	197	47.2	Referent
**Residential Status**			
Local	217	57.2	1.3 (0.8–1.9)
Non-local	359	47.1	Referent
**Duration of sex work (in years)**			
< 5	288	38.5	Referent
5–9	156	55.5	1.6 (1.0–2.5)
10+	133	66.4	2.6 (1.6–4.2)
**Number of clients per day**			
<3	333	37.5	Referent
3+	244	66.6	3.7 (2.5–5.5)
**Place of solicitation**			
Home-based	152	59.0	1.3 (0.8–2.2)
Brothel-based	146	54.6	0.7 (0.4–1.3)
Street-based	279	46.8	Referent
**Nature of solicitation**			
Independently	385	50.4	Referent
Through brokers/pimps	190	51.0	1.0 (0.6–1.7)
**District**			
Belgaum	208	63.5	2.3 (1.4–3.9)
Bangalore	369	41.6	Referent
**Total**	**577**	**50.5**	

1 **Risky sex:** Had unprotected sex with any partner and either had anal sex or consumed alcohol before sex or had concurrent sexual partnerships.

2 AOR: Adjusted Odds Ratio, CI: Confidence Interval.

$ Unweighted numbers.

## Discussion

This study found that a little more than half of the FSWs in Karnataka, India have engaged in risky sexual practices, measured using a summary measure of HIV risk and vulnerability. In addition, FSWs from Belgaum are more likely to engage in risky sexual practices than those from Bangalore. This may be the reason why HIV prevalence among FSWs in Belgaum is two times higher than among Bangalore-based FSWs [30]. Multivariate analysis indicated that engaging in risky sexual practices is positively associated with being older, currently married, in sex work for a longer duration, and having three or more clients a day. Post-hoc analysis suggests these groups of FSWs comprise 80% of the study population. Previous research studies conducted in India have also identified these factors as being associated with higher HIV infection among FSWs [30], [31]. The current study also notes that certain characteristics of FSWs are positively associated with one behavior, but negatively associated or not associated statistically with another behavior. For example, inconsistent condom use is higher among middle aged FSWs, concurrency is higher among those in the younger group, consumption of alcohol prior to sex is higher among older sex workers and finally, the practice of anal sex has no association with age of the sex worker. In this context, the summary measure of risky sexual practices could identify the most-at-risk group of FSWs, which can help program implementers to develop more definitive outreach strategies. Moreover, targeting these FSWs can help the program address both their vulnerabilities and risk behaviors simultaneously.

The study findings also suggest that more than two-thirds of FSWs have engaged in unprotected sex with their sexual partners. Evidence from research in India suggests that consistent condom use is considerably high with commercial partners [29], [32], [33], but has remained low with non-commercial partners [34]. The low level of condom use in non-commercial relationships can be due to the intimacy and trust involved in such relationships [35]; condom use in such relationships is most often perceived as a symbol of infidelity and fosters mistrust [36]. Further, the considerable proportion of FSWs involved in concurrent relationships coupled with high inconsistent condom use can lead to the transmission of STI/HIV infection at a much faster rate. Therefore, HIV prevention programs need to create greater awareness about the risk associated with non-use of condoms in non-commercial relationships with more emphasis on FSWs who have concurrent relationships. FSWs who are 25 years or older, currently married, and those entertain three or more clients a day should be given more attention than other sex workers to improve their condom use practices.

This study documented that 12% of FSWs had engaged in anal sex in Karnataka, which is considerably lower than findings from other research conducted in India among sex workers [10], [19], [37]. In these studies, around one in four FSWs reported ever engaging in anal sex with their sexual partners. The current study findings suggest that FSWs who have been in sex work for 10 years or more and have three or more clients a day are more likely to engage in anal sex than their counterparts. Similar findings are noted in another study conducted in East Africa [16]. Post-hoc analysis suggests that 62% of FSWs who have been in sex work for 10 years or more are 35 years or older. Evidence suggests that older sex workers get fewer clients to entertain than younger sex workers, [10] which may create a sense of fear among these FSWs regarding their survival in light of the reducing number of clients. Therefore, these FSWs succumb to the demands made by their clients which could be related to the type of sex, particularly anal sex, the place of sex or alcohol consumption [10].

This study found that more than half of the sex workers consumed alcohol prior to sex, which is similar to findings from another Indian study [14]. Empirical research has opined that alcohol use depends on the type of client with whom FSW have sex. FSWs consumed alcohol to enhance their enjoyment and involvement while having sex with regular clients, whereas with one-time clients, they consumed alcohol to become insensitive to take clients without emotional involvement [38]. The study findings also indicate that brothel-based FSWs and those getting clients through brokers/pimps were more likely to report alcohol consumption prior to sex than their counterparts. This could be due to the fact that alcohol is available within brothels and in nearby areas, and clients bring alcohol with them when they visit sex workers in these settings [38]. As these clients are accessible to sex workers only through a pimp or broker, structural interventions to increase awareness about the harm associated with alcohol use can be designed targeted at these stakeholders in sex work.

This study noted that a significantly higher proportion of sex workers in Belgaum than in Bangalore engage in risky sexual practices. In fact, the difference between these districts is more prominent in the practice of anal sex and alcohol consumption prior to sex than that in inconsistent condom use and concurrent sexual relationships. This difference could be due to the socio-cultural practices related to sex work practices in these districts. In Belgaum, the practice of sex work is concentrated in semi-urban and rural areas, characterized by the presence of traditional sex worker (Devadasi) and solicitation occurs primarily in non-street-based settings (home and brothel) [39], [40]; however, in Bangalore sex work is concentrated in urban areas and most FSWs solicit clients in street-based venues. Mapping data from these districts suggest that the number of sex workers in Belgaum is much less than in Bangalore [25], [26]. Further, FSWs in Belgaum are highly mobile, visiting districts in the nearby state of Maharashtra [39]. These factors either directly or indirectly affect the HIV risk behavior of FSWs as well as their clients. Therefore, these differences and contextual factors need to be taken into account while implementing HIV prevention programs in these districts.

Though the study findings are of utmost importance for HIV prevention programs and research, these findings must be interpreted in light of the following study limitations. First, the survey did not collect any biological information and hence, one cannot ascertain if the risk groups identified here also have higher STI/HIV infection rates. However, contemporary research studies that collected biological specimens suggest similarities in the risk group identified [30]. Second, previous research suggests that the prevalence of anal sex may have been under-reported, as the information was self-reported and the stigma associated with reporting such sensitive experiences is well recognized in many research studies [41]. Third, to measure concurrency, this study used the partner calendar method to gather information on duration of relationships with different sexual partners. Though this method provides more data, there may be recall bias/errors related particularly to the start and end dates of partnerships. However, previous research suggests that the estimates provided by this method of data collection can be considered reliable [42]. Fourth, data were collected in a cross-sectional survey and hence, the cause–effect relationship is difficult to establish. Fifth, there could be some extent of bias due to the use of the cluster sampling method to select hotspots. Despite these limitations in this study, the evidence from this study can guide policy makers in devising strategies for optimum outreach and service provision. Special prevention strategies need to be designed to increase risk perception about HIV among the most-at risk group of FSWs identified in this study.

In summary, this study identified four most-at risk groups of sex workers: 35 years or older, currently married, engaged in sex work for 10 years or more and have sex with three or more clients a day. HIV prevention programs should be able to cover more than 80% of FSWs if they target these sub-groups of sex workers. Special attention is needed in such programs at clinics to build the skills of FSWs on safe sex negotiation with clients. Further, the most-at-risk groups of sex workers identified are in disadvantaged life situations. Hence, efforts should be made to increase their earning from sources other than sex work through alternate livelihood mechanisms. Collectivization of FSWs is another important aspect that can bring these sex workers together in one place where they can be educated about safe sex practices. These initiatives also need to undertake awareness campaigns within sex work settings to educate both clients and FSWs on the need for safe sex practices. Advocacy with community stakeholders as well as with brothel owners and pimps can be another approach to reach these sex workers. Vulnerability to HIV risk through alcohol consumption can be reduced through risk reduction counseling and harm reduction measures. In addition, developing district specific intervention strategies that consider the prevailing socio-cultural norms in the district may yield better outcomes in HIV prevention programs.
